# Establishing community partnerships to maintain social connection and healthcare access during an emergency declaration: virtual veterans socials

**DOI:** 10.3389/fpubh.2025.1548618

**Published:** 2025-08-01

**Authors:** Jay A. Gorman, Elizabeth S. Chamberlin, Helen P. Hailes, Marsha Langer Ellison, Rachelle M. Calixte, Jessica Mack, John Smolinsky, Erin D. Reilly, Charles E. Drebing

**Affiliations:** ^1^United States Department of Veterans Affairs, VA Bedford Healthcare System, Bedford, MA, United States; ^2^Chobanian & Avedisian School of Medicine, Boston University, Boston, MA, United States; ^3^UMass Chan Medical School, Worcester, MA, United States

**Keywords:** social connection, loneliness, access to care, public-private partnerships, veterans, emergency preparedness, virtual support

## Abstract

This community case study reflects the transition of a community-based social intervention at a Veterans Affairs (VA) Medical Center in the northeastern United States (U.S.) from in-person to virtual gatherings during COVID-19 restrictions. Initially, the Veterans Socials program began in 2014 to address social support challenges of veterans who received care from VA, then evolved into community-based social groups delivered with and for the veteran community. These consist of veteran-led weekly meetups aimed at fostering social connection and reducing isolation. This evaluation explores the challenges and opportunities involved in shifting to a virtual platform, focusing on how community partnerships, including collaboration between private and government organizations, supported the continuity of this inclusive social support initiative. Leveraging these partnerships expanded access to health-related resources and services, while maintaining critical social networks. Findings include program-level data and three individual case studies that underscore the role of social support systems in mitigating loneliness and improving healthcare accessibility in times of crisis. This evaluation highlights how community partnerships were essential for sustaining social support, enhancing access to healthcare information, and disseminating vital information to the community during an emergency declaration.

## 1 Introduction

Efforts to limit the spread of the COVID-19 pandemic led to the unfortunate side effect of reducing social connections (1), and in this way, exacerbated another epidemic in the U.S., the ongoing epidemic of loneliness that has been a source of growing concern over the past two decades (2). Loneliness refers to the negative subjective experience of having a social network that is inadequate in quality and/or quantity (3), while social isolation refers to an individual’s limited social relationships, group memberships and infrequent social contacts (4). Two meta-analyses, including a combined 160 studies (*N* of 5.6 million), suggest that social isolation and loneliness increase the risk of early death by 29–32% (5, 6). Loneliness and social isolation can impair mental health and physical functioning (6, 7), predict depression severity and suicidality (8). One of the most prominent predictors of loneliness is the low frequency of high-quality contact with supportive friends and family (9, 10). Interventions that increase social connection, by enhancing the size and diversity of one’s social network, the roles and functions of relationships and the positive qualities of the relationships (2) can ameliorate the negative outcomes associated with loneliness (11).

Further, experiences of loneliness can create cognitive distortions whereby individuals perceive social interactions as more threatening and view their environment as fundamentally less safe (12). This altered perception can heighten the sense of personal vulnerability and increase beliefs about potential threat from others (13). Moreover, evidence indicates that increased feelings of being unsafe related to loneliness are significantly associated with unsecured firearm storage practices, which substantially elevates the risk for death by suicide (14, 15).

Interventions thwarting loneliness or social isolation may particularly be necessary for veterans, who may be vulnerable to loneliness across various age groups, due to challenges transitioning from the military and feeling disconnected from community networks (16–19). Among veterans who participated in the National Health and Resilience in Veterans survey 2019–2020 (*N* = 4,069; mean age = 62), 56.9% reported feeling lonely sometimes or more (20).

### 1.1 Virtual interventions to promote social connection

Historically, interventions that increase social connection have been based on in-person interactions, though a shift to online platforms has accelerated and gained acceptance across age groups (21–23). Healthcare policy changes during the COVID-19 pandemic and patients’ increased willingness to use telehealth services have made virtual interventions more acceptable and, at times, even essential to receiving care (24). Further, online social connections during the COVID-19 pandemic may have played an important role in maintaining health through mitigating loneliness (25).

During the COVID-19 pandemic, VA-led several telehealth interventions in the veteran population targeting social support and loneliness among veterans, with mixed results in loneliness. For example, VA CONNECT a telehealth cognitive-behavioral therapy and dialectical-behavioral therapy skills group that integrated peer support did not show significant changes in loneliness over 10-sessions, but significantly reduced perceived stress (26). A 12-session intervention CONNECTED, showed peer-facilitated group sessions were both feasible and preliminarily effective, showing reductions in social isolation and increased perceived social support (27). Another initiative, Compassionate Contact Corps, matched veterans with trained volunteers for weekly supportive phone calls, providing a scalable approach with goals of reducing loneliness and fostering social connection (28).

Naslund et al. (29) proposed a conceptual model for online peer-to-peer social connection with persons experiencing mental health challenges. This model addressed potential benefits, including opportunities to challenge stigma, increase consumer activation, and access to mental and physical wellbeing interventions while engaging with peers online. It also identified possible risks of using online communities to engage patients, including feeling more uncertain about one’s diagnosis or condition. In addition, the online disinhibition effect can further amplify risks by increasing the likelihood of aggressive or derogatory interaction among users (30). To effectively implement online peer-to-peer support interventions, the benefits, risks, and technological barriers must be continually explored, evaluated, and addressed to further understand best practices in virtual platforms for social interventions.

### 1.2 Public-private partnerships that can support social connection

Leveraging public-private partnerships can capitalize on the shared goals of government and private organizations to better serve veterans and improve the standard of care for veterans and their families (31). Collaborations through public-private partnerships are advancing across multiple areas including suicide prevention (32), support for transitioning service members and coordinating care (33), education (34), employment (35), information access and dissemination (36). As social relationships become more prioritized in public health policy, the urgency to explore social interventions intensifies (37). Non-medical interventions aimed at enhancing social connection for veterans, often supported by community volunteers and private organizations have also gained traction. TogetherWeServed.com, a U.S. military and veteran online community, launched a veteran Buddy Link on its platform to help connect veterans seeking camaraderie connect online with others who live nearby, registering over 2,200 users looking for a local buddy within the first month of launch (38). Veteran and military-affiliated gaming communities (e.g., Regiment Gaming, Warrior GMR Foundation, and Stack Up) have grown in popularity (39) and may have the potential to support social connection initiatives and public health goals (40, 41).

### 1.3 Veterans socials

In 2014, peer specialists (PSs) employed by the U.S. Department of Veterans Affairs developed and co-hosted Veterans Socials (VSs) in communities around VA facilities. These were developed through community partnerships between VA, veteran serving organizations and veteran community leaders over 5.5 years. PSs worked with community leaders to initiate weekly VSs, which evolved based on the interests and preferences of attendees (e.g., coffee meetup, bowling leagues, gaming clubs, volunteer groups). Further, VSs offered an inclusive approach to community building, often incorporating military affiliated family members, caregivers, and supportive others in social activities. This flexible, community-based social intervention was designed to offer a low barrier to entry, with no attendee requirements. Essential elements of the VSs included:

1. VS is held in a community location other than a VA facility.
2. VS is open to all military-affiliated and supportive others who wish to join.
3. Attendance is not entered in the veterans’ medical records.
4. VS is held at least once per month (in-person or virtually).
5. The primary objective is to foster social interaction in the community.
6. Attendee preferences drive the format, content, and process of the VS.
7. The host presents opportunities for attendees to socialize outside of the VS.
8. The host has materials (e.g., health and community activity information) available to assist attendees in choosing to pursue VA and/or community resources and services.

VSs were founded on strong partnerships between veterans and their local communities. In collaboration with community leaders, the creation of VSs followed guidance that involved designing, organizing, and implementing events in partnership with attendees and local organizations (42). These efforts employed a multi-channel, community-based approach to recruiting attendees, relying entirely on no-cost recruitment strategies. VA PSs and community leaders advertise in local businesses (e.g., grocery stores, health centers, restaurants, community centers), veteran organizations and in VA hospitals, as well as through social media, and word-of-mouth campaigns. As a result, VSs typically gain attendees progressively over a 4- to 6-month period. Veteran leaders and PSs often collaborated to initiate and co-host VS, with the aim of developing sustainable, regularly scheduled (e.g., weekly or monthly) veteran gatherings in the community (43). A positive social group culture within VSs is demonstrated when hosts and attendees actively discuss community events, share personal stories including stories that highlight the importance of seeking help when needed, and exchange information on community and VA resources. The overarching goal was to build community-based social support systems by harnessing the power of veteran-to-veteran connections.

### 1.4 Community partnerships

Collaborative community-level interventions are foundational for long-term and sustainable community impact (44). Moreover, in times of crisis social infrastructure bolsters community resilience and drives the collective ability of a population to withstand and recover from crises (45). Community partnerships were initiated and maintained through an active engagement strategy led by peer specialists from the Veteran Community Connection Team, with support from VA hospital leadership. This team regularly participated in community meetings, joined initiatives led by veteran-serving organizations, and collaborated with local collaboratives in surrounding areas. Over time, strong relationships were formed between VA staff, town leaders, state veterans services, non-profit veteran organizations, and local businesses. These partnerships were nurtured and sustained through ongoing contact at in-person VSs, which provided a consistent platform for collaboration and relationship-building. In March of 2020, restrictions associated with the COVID-19 pandemic (46) led to the abrupt cessation of all 28 in-person VSs across two VA medical center catchment areas. Community partnerships between neighborhood organizations, businesses, key leaders, and local government facilitated the rapid development and implementation of Virtual VSs. Through three cases studies, this report explores the process of developing and implementing Virtual VSs, opportunities for increased accessibility, and benefits of collaborating with community partners.

## 2 Context

### 2.1 Process of adapting VS

PSs adapted in-person VS to a virtual format. The PSs advertised the Virtual VS by contacting VS hosts, veterans who subscribed to the monthly VS newsletter, local veteran organizations (e.g., American Legion, The Veterans of Foreign Wars, Disabled American Veterans), state and city veteran services, veterans service officers, local business, the organizations that had provided space for the in-person VS, and the local Veteran Affairs (VA) medical center communication channels. Further, the Virtual VS was advertised on community-run VS Facebook pages, flyers in local VAs, and a word-of-mouth campaign that encouraged veterans to spread information about the Virtual VSs.

The PSs who co-hosted Virtual VSs examined various virtual platforms such as Skype, Zoom, Webex, and Microsoft Teams. They spent time on each platform and considered who the audience would be and what attendees would need. Considerations included: (1) having a virtual platform that would allow veterans to call in with a phone as well as a computer; (2) making the Virtual VSs feel comparable to an in-person VS by employing a platform that used video and a share screen function; and (3) using a platform that was easy to navigate to allow people with varying levels of technical abilities to join.

### 2.2 Attendance and participation

Unlike in-person VSs, Virtual VSs required more facilitation demands from the host. Virtual platforms also eliminated side conversations, which often occurred during in-person VS. The Virtual VS host would lead the conversation and monitor the chat as another form of communication. The Virtual VS host would also assist attendees if they had challenges using the technology during and before the Virtual VS. They also discussed ways of making it clear when one was done speaking, as social cues were more difficult to interpret.

The Virtual VSs began in late March 2020 and continued for 14 months, during which a single VA facility—working in partnership with the community—co-led 234 virtual events, resulting in 1,226 attendee engagements (non-unique). For program evaluation purposes, anonymous information was collected by hosting PSs during Virtual VSs. Initially, these Virtual VSs were held daily, Monday through Friday, with two hosts typically in attendance. The average number of attendees per Virtual VS from March to July 2020 was seven. However, starting in August 2020, attendance began to decline, with an average of 4.5 attendees per session from August 2020 to March 2021. In response to this decreased attendance, the frequency of Virtual VSs were reduced to 3 days per week beginning in October 2020. The downward trend in participation continued, with attendance further declining to an average of 3.5 attendees per session during the final 2 months (April and May 2021). Consequently, Virtual VSs were mostly discontinued after May 2021, primarily due to veterans’ expressed preferences for attending in-person socials. This shift in format reflects the program’s responsiveness to changing attendance patterns and participant preferences throughout the evaluation period.

The content of the initial Virtual VS primarily focused on practical matters, including familiarization with the virtual platform, dissemination of COVID-19-related information, and discussion of available resources for veterans in need. Hosts occasionally prepared specific topics for discussion, such as self-care strategies, locations of food pantries, financial assistance options, and community resources. However, as regular attendance patterns emerged, the nature of these interactions evolved. Conversations transitioned from host-directed discussions to more attendee-driven dialogues, reflecting an organic development of community within the virtual space.

## 3 Case studies

This manuscript reflects a description of the Virtual VSs from the perspectives of the VS hosts. Three Virtual VS hosts were interviewed to provide three intrinsic case studies that describe phenomena for veteran participants in Virtual VSs during the COVID-19 pandemic. This approach aligns with the intrinsic case study method, which seeks to understand a particular cases due to their unique context or features (47). Three case studies are presented from the viewpoint of the Virtual VS hosts, who were interviewed for this information to allow these VS experts to select and describe case(s) that would provide unique insights into the program and, in doing so, illustrate implementation, impact, and lessons learned. This also allowed for a more naturalistic understanding of veteran experiences within the Virtual VS process and setting, without causing any additional burden of obtaining information directly from veterans during a particularly challenging time. Each case highlights features of veterans’ experiences learning about and engaging with the Virtual VS.

### 3.1 Case 1—Virtual veterans socials: facilitated social support in the absence of in-person options

Betty (name changed), a White female Gulf War veteran in her 60s, had participated in an in-person VS regularly, often assisting the host and bringing snacks for the other attendees. Betty was considered an essential worker, a designation that required her to be present on-site during hours that conflicted with the Virtual VS. As a result, she was unable to attend the Virtual VS regularly throughout the COVID-19 pandemic. As capacity limits on businesses were relaxed, she began attending the few local in-person VSs and gradually decreased her attendance in the Virtual VS.

However, in the fall of 2020, she contracted COVID-19 and was homebound for weeks. At that time, Betty participated in a Virtual VS four times per week and utilized the platform’s video conferencing capability to interact. After that experience, she took on more responsibilities in the Virtual VS and conducted buddy checks, which consisted of a brief phone call to veterans with whom she had formed relationships and had not attended the VS recently. Overall, she viewed the Virtual VS as a beneficial “option” when “you need that support.” Following this experience, she chose to participate in additional social and health opportunities through virtual platforms, including a virtual art group and VA group therapy sessions using video conferencing technology—a pattern that suggests reduced isolation.

### 3.2 Case 2—Virtual veterans socials: facilitated increased engagement

Connor (name changed), a White male Vietnam-era veteran in his mid-70s, was introduced to the VS at his local senior center. He attended a VS for approximately 1 year before the virtual adaptation, typically attending one or two events per month, including a local bowling group that evolved from a VS. The veteran’s initial social engagement at the VS was limited. Connor attended infrequently, and when he did attend, he had limited interaction with others and disclosed little about himself, his feelings, or his personal history.

As COVID-19 restrictions were put in place and social events were canceled, a PS at the local VA, who was familiar with Connor through in-person VSs, encouraged him to attend the Virtual VS and attempted to help him with connecting virtually. Connor expressed apprehension and reluctance to navigate the virtual platform; however, he was amenable to using his phone to call into the Virtual VS. Connor reported he could no longer have visitors in his home, including his grandchildren, due to the pandemic. He also reported feeling anxious about leaving his home due to underlying medical conditions that made him vulnerable to COVID-19 and lacked social connection because of the safety measures he was taking to protect himself; the Virtual VS offered a platform for social connection.

Connor attended the Virtual VS daily. While his other social events were canceled, the Virtual VS provided a reliable outlet for social engagement, and his increased attendance suggested reduced loneliness; moreover, he began to share more personal information about himself, such as his financial difficulties, which prompted other attendees to offer information and community resources to support him. He reported to the Virtual VS host that he was enrolling in the local VA to assist him with his other psychosocial challenges. When Connor decided to enroll in VA services, he saved money by switching his medical care, substantially reducing his medication costs, and allowing him to more effectively manage his other healthcare needs.

### 3.3 Case 3—Virtual veterans socials: facilitated access to care

The final case illustrates how Virtual VSs can lead to healthcare utilization by enhancing awareness of one’s own needs, as facilitated by a caring social group. In this case, Dave (name changed), an 80-year-old White male Vietnam-era veteran, was influenced to seek needed healthcare services. This veteran had attended three different VSs for several years. During the COVID-19 pandemic, the veteran received information about Virtual VSs from a PS at his local VA and was encouraged from PSs whom he had met through in-person VSs. Dave was adept with technology but preferred using his phone to call into the Virtual VS rather than using the virtual platform. He attended the Virtual VSs approximately 2 days per week.

During his participation in the Virtual VSs, he noted that he had a wound that he treated himself and that it was becoming increasingly painful. Dave lived alone and had not told anyone about his injury, nor had he sought medical treatment. Other attendees discussed that he had reported this pain on a previous Virtual VS and asked him to show them the wound on camera. They persuaded him to seek immediate treatment. Dave was enrolled in VA services but had not considered scheduling an appointment. With encouragement from the other attendees and a subsequent phone call from a PS informing him of procedures for accessing urgent care services, Dave received treatment for his injury. Dave updated attendees at the Virtual VS and reported staying in the hospital for multiple days to treat the infection. He was grateful that he received encouragement and support from his peers, as it resulted in him accessing vital healthcare services and getting treatment for a potentially life-threatening infection. He has shared his story with other veterans to encourage them to utilize VA healthcare resources.

## 4 Discussion

Many lessons were learned during the transition from in-person to Virtual VSs. One is the value of Virtual VSs for increasing access for individuals who might not otherwise engage with services and supports. Another is the importance of community partnerships in facilitating social connection and the diffusion of information during community crises.

While in-person activities and services that were suspended due to COVID-19 pandemic restrictions have resumed, the same groups of individuals who faced obstacles to in-person care before the pandemic remain and benefit from the continued offering of flexible, hybrid, and virtual services (48). Furthermore, situations that can cause disruptions to in-person programming continue, with other pandemics increasingly likely (49), natural disasters, and extreme weather events such as heat, flooding, and drought. Additionally, health-related concerns (e.g., mobility issues or being immunocompromised), pose barriers to travel or participation in community events, and may continue to create obstacles to in-person care and connection for large numbers of people (50, 51). For all these reasons, the ongoing provision of virtual interventions and enhancement to community partnerships for clinical and non-clinical services is critical to meet the needs of veterans – especially during community, state, national, and global crises.

### 4.1 Impact of virtual veterans socials

Each case exemplifies key benefits of the Virtual VSs format, namely the facilitation of continued social support in the absence of in-person options, enhanced awareness of one’s own needs, and readily available access to information and care options. The Virtual VSs experiences of attendees reported to and observed by Virtual VS host illustrate the benefits of these virtual gatherings, particularly during periods of heightened isolation such as the COVID-19 pandemic. In Case 1, Betty, demonstrates how attendees can assume meaningful, purposeful, and empowering roles in virtual social groups. By conducting buddy checks with fellow participants, Betty not only contributed to a supportive environment but also exemplified the potential for peer leadership within virtual communities. Moreover, Betty’s engagement with the Virtual VSs appears to have encouraged her exploration of additional virtual activities, including healthcare treatment, suggesting that these gatherings may serve as a gateway to broader utilization of supportive services.

Connor’s case underscores the evolving nature of engagement that Virtual VSs can facilitate. Initially a sporadic and reticent attendee, Connor’s attendance became more consistent and open following the transition to virtual formats. The virtual environment provided a safe and reliable platform for social interaction, ultimately enabling Connor to disclose personal challenges—such as financial difficulties—within the group. This openness led to peer-driven resource sharing and support, with positive outcomes in financial, social, and health domains. Financially, Connor gained access to community resources and cost-saving VA healthcare options. Socially, increased participation offered a crucial outlet for interpersonal connection during pandemic-related restrictions. Furthermore, Connor’s engagement encouraged his enrollment in local VA care, highlighting the potential of Virtual VSs to foster supportive environments where veterans feel comfortable sharing challenges and accessing needed services.

Dave’s experience further illustrates the potential health-related benefits of regular Virtual VS participation. The frequency of these gatherings enabled attendees to monitor changes in Dave’s well-being over time, while the virtual platform provided ongoing access to social support and reminders about available healthcare resources. Encouragement from peers motivated Dave to seek care, demonstrating how Virtual VSs can facilitate access to healthcare for veterans who may otherwise underutilize needed services. Additionally, sharing Dave’s success with peers may reinforce his continued use of health services and motivate others to seek care, amplifying the positive impact of these virtual gatherings.

Overall, these case examples collectively demonstrate that Virtual VSs have the potential to serve as a vital platform for peer support, resource sharing, and health service utilization among veterans. Further, Virtual VSs served a dual purpose: they provided PS hosts with a channel to reach potentially isolated veterans who might not otherwise access services while simultaneously offering veterans an opportunity for social connection.

### 4.2 Technological implementation and accessibility

During the COVID-19 pandemic, virtual interventions became common; for the first time, we *all* experienced a collective, extended obstacle to in-person services. The case examples above highlight one intervention’s successful transition from in-person to virtual, not only continuing to provide veterans with social support and access to services and information. The Virtual VS was delivered via mobile phones, computers, and audio-only options, allowing accessibility for attendees with varying levels of technological proficiency. Virtual VS hosts assisted attendees prior to the meet-up to gradually build attendees’ capacity to access the Virtual VS on their own. The platforms used required stable internet connections or data plans that can support usage, which can be challenging for those in rural areas or areas with less internet coverage, wireless signal, or other general resources (e.g., phone, laptop). The platforms (i.e., Zoom, Teams, and Webex) used to engage attendees consisted of features like video conferencing and screen sharing, helped create an engaging and interactive experience for participants who could access chat, send images, reactions, or emojis to one another. VS hosts selected specific virtual platforms based on their comfort, attendee preferences and barriers associated with the platform (e.g., software update frequency, web browser compatibility) (41). The number of Virtual VS engagements during COVID-19 restrictions indicates that, in certain situations, virtual interventions can serve as a pragmatic community approach to increase access to information and reduce loneliness. The veteran population is, on average, older (age 65) than the general population than the general population (age 39) (52, 53). Although, there is a common myth that older adults are not interested in or capable of using technology (54), some veterans who appeared to receive the most benefit from the Virtual VS were older. However, it is also possible that older veterans may have had risk factors that made them more susceptible to loneliness than others (55) and Virtual VS contact may have appeared to benefit them in ways that may have not been beneficial or were disliked by others without those risk factors. Moreover, some of these older individuals were isolated and had a higher risk of becoming critically ill from in-person socializing during the COVID-19 pandemic (56).

Overall demand for the Virtual VS decreased substantially as in-person socializing gradually returned over the course of the COVID-19 pandemic, indicating many individuals’ preference for in-person support. Moreover, VS hosts reported the VS “felt different than in-person…and not the same” suggesting that virtual interactions may not have been as rewarding as in-person VS interactions. Although many in-person VS resumed after restrictions were lifted, it is also possible that some Virtual VS attendees decided not to return to in-person VS if they did not find the intervention valuable, useful, or for other reasons that prevented them from attending. The Virtual VS is not a typical virtual intervention; it is designed to be a low-obstacle, highly accessible social support intervention, however barriers remain. Any military-affiliated or community supporter can attend a VS and there is no requirement that attendees engage with the VA healthcare system. While veterans who are enrolled in VA care have expressed a willingness to engage in VA services to reduce social isolation (27), there are many unenrolled veterans, due to the stigma of seeking care (57), mistrust of VA or medical establishment (58), or distance they would need to travel (59), who do not seek help within VA. The VSs were intended to be an inclusive social intervention accessible to all veterans, regardless of VA enrollment status. As of 2025 there were 178 known VS across 26 U.S. states and territories. The expansion of VSs to incorporate virtual options as needed reflects a commitment to ensuring veterans have resilient pathways to access healthcare information and social support systems in their communities.

## 5 Conclusion

The COVID-19 pandemic heightened feelings of loneliness and social isolation, compounding an issue already recognized as an epidemic in the United States (60). As such, there persists a crucial need for resilient and accessible interventions addressing these concerns. The VS virtual adaptation was catalyzed with the collaboration between government, community organizations, businesses, and institutions. Going forward, Virtual VSs may serve as a valuable addition when in-person gatherings are not feasible, effectively supplementing—rather than replacing—traditional in-person VSs. The successful adaptation of the VS format to a virtual environment highlights its potential as a flexible tool for sustaining veteran engagement and support during periods of adversity. Additional evaluations of virtual and non-virtual VSs, including obtaining feedback directly from veteran participants, could further highlight ways to increase access to and support from VS programming. Overall, this community case study demonstrates that community partnerships forged through VSs have the potential to serve as crucial conduits for rapidly disseminating information and bolstering social support during emergencies.

### 5.1 Acknowledgment of conceptual or methodological constraints

The evaluation has several limitations. The community case study design is limited in nature and may not extend to a broader population outside of the community evaluated. Additionally, the evaluation’s scope was intentionally limited to avoid disrupting the intervention and programming during an emergency declaration. Furthermore, the participant feedback described in these case studies may have been influenced by response bias or demand effects, as only Virtual VS hosts were interviewed for this evaluation, underscoring the need for more rigorous studies to gain a clearer understanding of program benefits. Overall, more systematic investigation is required to evaluate the impact of VSs on social connection. Ongoing studies are currently examining how this intervention affects loneliness and social isolation among participants.

## Data Availability

The raw data supporting the conclusions of this article will be made available by the authors, without undue reservation.
